# Glucocorticoids Are Not Associated with Bone Mineral Density in Patients with Polymyalgia Rheumatica, Giant Cell Arteritis and Other Vasculitides—Cross-Sectional Baseline Analysis of the Prospective Rh-GIOP Cohort

**DOI:** 10.3390/cells11030536

**Published:** 2022-02-04

**Authors:** Andriko Palmowski, Edgar Wiebe, Burkhard Muche, Sandra Hermann, Christian Dejaco, Eric L. Matteson, Frank Buttgereit

**Affiliations:** 1 Department of Rheumatology and Clinical Immunology, Charité—Universitätsmedizin Berlin, Corporate Member of Freie Universität Berlin and Humboldt-Universität zu Berlin, 10117 Berlin, Germany;; 2 Department of Rheumatology and Immunology, Medical University Graz, 8036 Graz, Austria;; 3 Rheumatology Service, South Tyrol Health Trust, 39031 Bruneck, Italy; 4 Departments of Internal Medicine and Health Sciences Research, Division of Rheumatology and Division of Epidemiology, Mayo Clinic College of Medicine and Science, Rochester, MN 55905, USA

**Keywords:** glucocorticoids, vasculitis, polymyalgia rheumatica, giant cell arteritis, bone density, osteoporosis

## Abstract

Background: Glucocorticoids (GCs) can cause osteoporosis (OP). Prior observational research on bone density and the effects of GCs in polymyalgia rheumatica (PMR) and vasculitides is scarce and inconclusive. Methods: Rh-GIOP is a prospective cohort study of bone health in patients with inflammatory rheumatic diseases. In this cross-sectional baseline analysis, we focused on patients with PMR and different forms of vasculitides. Multivariable linear regression was used to model the effect of current and cumulative GC intake on the minimum T-score at any site (mTs; at either lumbar spine or hip), with comprehensive adjustment for confounders. In separate models, GCs were modelled both as continuous and categorical predictors. Sensitivity analyses, stratifying by measurement site and disease, were conducted. Results: A total of 198 patients, with a mean age of 67.7 ± 11.4 years and a mean disease duration of 5.3 ± 6.3 years, were included. Most patients suffered from PMR (36%), giant cell arteritis (26%) or granulomatosis with polyangiitis (17%). Women comprised 66.7% of the patients, and 87.4% were currently taking GCs. The mean CRP was 13.2 ± 26.1 mg/L. OP diagnosed by dual energy X-ray absorptiometry (DXA) (T-score ≤ −2.5) was present in 19.7% of the patients. While 88% were taking vitamin D supplements, calcium supplementation (4%) and treatment with anti-resorptive agents (17%) were relatively infrequent. Only 7% had a vitamin D deficit. Neither current (*β*(continuous model) = −0.01, 97.5% CI –0.02 to 0.01; *p*(all models) ≥ 0.49) nor cumulative (*β*(continuous model) = 0.01, 97.5% CI −0.04 to 0.07; *p*(all models) ≥ 0.35) GC doses were associated with mTs in any model. CRP was not associated with mTs in any model (*p*(all models) ≥ 0.56), and no interaction between CRP and GC intake was observed (*p* for interaction(all models) ≥ 0.32). Across all analyses, lower body mass index (*p*(all models) ≤ 0.01), history of vertebral fractures (*p*(all models) ≤ 0.02) and proton-pump inhibitor intake (*p*(all models) ≤ 0.04) were associated with bone loss. Sensitivity analyses with femoral neck and lumbar spine T-scores as dependent variables led to similar results as the analysis that excluded patients with PMR. Conclusions: In this cohort of PMR and vasculitides, we found a similar prevalence of OP by DXA to the overall elderly German population. Vitamin D supplementation was very common, and vitamin D insufficiency was less frequent than expected in Germans. There was no association between current or cumulative GC intake, CRP and impaired bone density. Proton-pump inhibitors seem to be a major, but somewhat neglected, risk factor for OP and should be given more attention. Our findings require confirmation from longitudinal analyses of the Rh-GIOP and other cohorts.

## 1. Introduction

Patients suffering from inflammatory rheumatic diseases (IRDs), such as vasculitides, are prone to bone loss and fragility [1]. In giant cell arteritis (GCA) for example, the fracture risk is increased by 67% compared to matched controls [2].

Inflammation itself impairs bone health. While the exact process has not yet been fully elucidated, pro-inflammatory cytokines (e.g., IL-1, IL-6 and TNF-alpha) have been found to impede bone formation [3]. At the same time, these cytokines also enhance osteoclastogenesis [3]. In patients with IRDs, modified bone remodeling, increased resorption and insufficient bone formation lead to both localized and generalized osteoporosis (OP).

Glucocorticoids (GCs) are commonly used drugs in the treatment of vasculitides. Indeed, medical guidelines underline the role of GCs as a mainstay in the management of, e.g., large vessel vasculitis [4], ANCA-associated vasculitis [5] and polymyalgia rheumatica [6] (PMR). They have unparalleled anti-inflammatory and immunomodulatory effects, mediated by both genomic and non-genomic modes of action [7]. However, GCs have a variety of adverse effects, such as weight gain, hypertension and OP. GCs directly inhibit osteoblasts and spur osteoclasts [1].

Until now, few studies have assessed the role of GCs in the bone health of patients with vasculitis. Using comprehensive data from the ongoing Rh-GIOP cohort and bone mineral density measurements, and taking into account numerous protective and risk factors, we examined associations of GC intake and bone mineral density in patients suffering from vasculitides and polymyalgia rheumatica (PMR).

## 2. Methods

Rh-GIOP (registered with ClinicalTrials.gov; identifier NCT02719314) is an observational cohort study investigating bone health in patients suffering from IRDs and psoriasis. It is a prospective single-center study conducted at a university hospital and tertiary care center (Charité—Universitätsmedizin Berlin), including both in- and outpatients. The institutional ethics committee approved Rh-GIOP (EA1/367/14). Every patient provided written informed consent. Data collection started in July 2015 and is ongoing. A patient representative has been involved in this study from the outset in order to help identify relevant research questions and outcomes, and to represent the interests of patients concerning the study.

Inclusion criteria are age ≥ 18 years, diagnosis of an IRD or psoriasis, current or previous treatment with GCs and eligibility for a structured diagnostic OP procedure as recommended by the “Dachverband Osteologie” (German Umbrella Association for Osteology) [8]. Pregnant or breastfeeding women are excluded, as are patients unable to provide informed consent.

Data collected at each visit include demographics and general information, details of current and previous GC therapy, features of the underlying disease and a variety of bone-relevant variables, such as calcium and vitamin D supplementation, family history of osteoporosis and specific laboratory markers. A detailed list can be found in Table S1. Bone density is measured at the right and left femoral neck and at the lumbar spine with a Lunar Prodigy (GE Medical Systems Lunar Corporation, Madison, WI, USA) bone densitometer by dual X-ray absorptiometry (DXA). DXA results are presented as a T-score (Ts), which equals the standard deviation (SD) from the mean of an average healthy 30-year-old adult. According to the WHO definition, Ts ≥ −1.0 were considered normal, <−1 to >−2.5 osteopenic and ≤−2.5 osteoporotic [9].

All data are entered into a Microsoft Access database (Microsoft Corporation, Redmond, WA, USA). The database was programmed by Medikadat Limited (Leverkusen, Germany). The current study includes all patients with polymyalgia rheumatica (PMR), giant cell arteritis (GCA) and other vasculitides. For data analysis, cross-sectional data from the baseline visit were used.

Patient characteristics are presented as mean and standard deviation (SD) or n (%). The minimum Ts (mTs) of any of the sites measured in each patient was chosen as the dependent variable in all analyses (as the diagnosis of OP by DXA depends on the lowest measured Ts at lumbar spine, femoral neck or total hip). Current and cumulative GC dose were also categorized into quartiles. To better understand the effects of low-dose GCs (in contrast to no GCs), we formed a group with 0 mg/d current intake followed by a group of >0 mg/d to the 25th percentile.

First, scatter plots and boxplots of mTs and GC intake were created for visual inspection of potential associations. Then, in separate analyses, GCs (both current and cumulative doses) were modelled both as continuous and categorical predictors. The effects of current and cumulative GC doses (independent variables) on the mTs (dependent variable) were assessed in multivariable linear regression models with comprehensive adjustment. Table 1 presents an overview of the regression models. Patients with 0 mg/d current and <1.5 g cumulative prednisolone equivalent dose formed the reference group for categorical GC modelling.

We adjusted for known and highly suspected confounders (see below) instead of using stepwise selection methods [10]. As potential confounding variables (Table 2), we mainly included those used to predict the risk of an osteoporotic fracture (FRAX tool: https://www.sheffield.ac.uk/FRAX/ (accessed on 29 December 2021)) and those that were found to be significantly associated with mTs in the first cross-sectional analysis of the overall Rh-GIOP cohort (manuscript in preparation). Additionally, we adjusted the models for C-reactive protein (CRP) levels as a surrogate measure for inflammation. First-order interactions were included as well. Adjusted R² values are presented for judging model fit. Hereafter, “mg/d prednisolone equivalent” is referred to as “mg/d” for readability.

We excluded patients with early disease (<3 months) from inferential analyses and graphics because bone loss (be it due to inflammation or drugs) requires longer periods of time to be captured by DXA. Of note, some patients received very high GC dosages (i.e., >100 mg/d) at baseline as short-term induction therapy to treat flare. Rosner’s outlier detection test was applied to confirm such patients as formal outliers [11]. As such strong outliers might bias regression models, these patients were excluded from analyses in which current GC dose was the main independent variable and continuously modelled.

We conducted the following sensitivity to underpin our findings:

(1) Excluding patients with PMR;
(2) Lumbar spine Ts as dependent variable;
(3) Right femoral neck Ts as dependent variable;
(4) Left femoral neck Ts as dependent variable.

Multiple imputation by chained equations with ten replications was applied to handle missing data (about 5%). The two-sided significance level α was set at 0.05. R software (version 3.6.1) with package MICE [12] was used for statistical analysis.

## 3. Results

**Figure 1:**
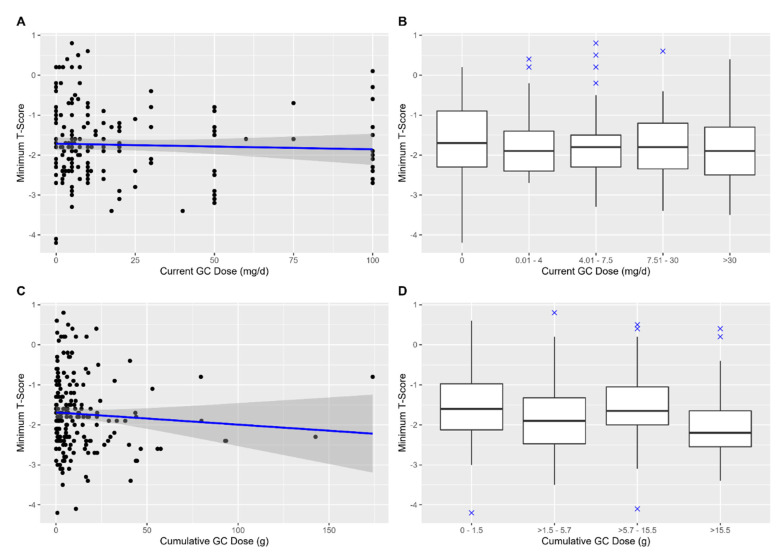
Scatter plots (**A**) and (**C**) and boxplots (**B**) and (**D**) of minimum T-scores and glucocorticoid (GC) intake. All plots: patients with a disease duration of <3 months were excluded. Plot A: patients with a current dose of >100 mg/d were excluded. In (**A**) and (**C**), the straight blue line represents a hypothetical regression line, accompanied by its 95% confidence interval (dark grey shadow).

The study enrolled a total of 198 patients (mean age 67.7 ± 11.4 years) with PMR, GCA and other vasculitides (Table 3). The mean disease duration was 5.3 ± 6.3 years, 66.7% were women and 87.4% were currently taking GCs with a mean dose of 30.8 ± 67.5 mg/d prednisolone equivalent. OP diagnosed by DXA was present in 19.7% of the patients. The mean CRP levels were 13.2 ± 26.1 mg/L. In total, 33% had a history of fractures due to inadequate trauma and 10% had a history of vertebral fractures. Scatter plots and boxplots depicting mTs by GC intake are shown in Figure 1.

In the adjusted analyses (Table 4), current GC intake was not associated with mTs, regardless of whether GC intake was continuously or categorically modelled (all: *p* ≥ 0.49). Furthermore, no negative associations between cumulative GC doses and mTs were found in any model (all: *p* ≥ 0.35).

CRP levels were not associated with mTs in any adjusted model (all: *p* ≥ 0.56), and we found no evidence of interaction between CRP and GC intake (all: *p*(interaction) ≥ 0.32). The following variables were significantly associated with mTs in all the adjusted models: body mass index (BMI; slope *β* (all models): 0.05 to 0.06; *p* < 0.01), prior vertebral fractures (estimated difference compared to “no prior vertebral fractures” (all models): −0.6 to −0.9; *p* ≤ 0.02), and intake of proton-pump inhibitors (estimated difference compared to “no proton-pump inhibitor intake” (all models): −0.3 to −0.4; *p* ≤ 0.04). In other words, for every unit of BMI increment, the mTs was increased by approximately 0.05 to 0.06, while the mTs was decreased by approximately 0.3 to 0.4 in patients taking PPI.

The current or cumulative GC dose was not associated with bone density in any sensitivity analysis (Table 5).

## 4. Discussion

In this study, cumulative GC dose and current GC dose were not associated with mTs in patients suffering from PMR, GCA and other vasculitides, following comprehensive adjustment for confounders. These findings were underpinned by sensitivity analyses stratified by measurement site, and were similar in patients with vasculitides (excluding those with PMR). Lower BMI, history of vertebral fractures and proton-pump inhibitor (PPI) intake were consistently linked with lower mTs.

Our findings call into question the idea of GC-*induced* OP in vasculitis. Yet, OP in vasculitis might be GC-*associated* (i.e., coincident) rather than GC-*induced*. Other risk factors seem to govern the overall risk of impaired bone density, namely, low BMI and PPI intake. As all the patients in our cohort are treated at a tertiary care university hospital, they might receive advanced treatment options for their underlying disease, which, in turn, could reduce unnecessary GC treatment and benefit the bone. Interestingly, only 18% received specific anti-osteoporotic treatment, despite a high number of patients being treated with GC dosages >7.5 mg/d. The vast majority of patients received vitamin D supplementation, leading to a low number of patients with a deficiency (7%)—in Germany, about 32% of adults are estimated to have a vitamin D deficiency [13]. Surprisingly, the prevalence of OP in our cohort was similar to overall (unselected) German populations of similar age [14,15].

In a recent meta-analysis, GC-related bone loss in chronic inflammatory diseases (mean GC dose 9.3 mg/d) in the first year of the treatment was also found to be less severe (spine: −1.7% of baseline bone density; femoral neck: −1.3%) than that measured in patients receiving higher-dose GCs in transplantation (mean dose: 15.7 mg/d; spine: −3.6%; femoral neck: −3.1%) [16].

Prior research investigating the effect of GCs on bone density in vasculitides is scarce. In Takayasu’s arteritis, Mo et al. recently reported that affected patients had lower bone mass than matched controls, underlining the fact that bone loss is a general occurrence in vasculitides, but medication use was not assessed [17]. Osteoporosis is a well-known comorbidity of GCA. A study from 2015 reported a risk ratio of 2.9 in patients with GCA compared to controls [18]. In PMR and GCA, Paskins et al. found a significantly increased overall occurrence of fractures (by about 63% and 67%, respectively) compared to matched controls [2]. Among patients with PMR, GC intake was not associated with an increased risk of fracture at lower dosages (<7.5 mg/d) in adjusted analyses [2], whereas in GCA, the fracture risk was increased at dosages ≥5.7 mg/d in adjusted analyses [2]. Unfortunately, they did not assess the effects of different dosages in the low-dose range on fracture occurrence. This publication from Paskins et al. referred to patients diagnosed between 1990 and 2004, so one has to bear in mind the substantial changes in treatment routines over time. Nowadays, the initial doses of glucocorticoids are substantially lower than 20–30 years ago, and rheumatologists broadly use preventative measures, such as prescribing calcium and vitamin D3—at least when higher dosages of GCs are used. Every guideline for GC-associated/-induced OP recommends those supplements. In our cohort, the number of patients with low or very low vitamin D levels was surprisingly low, while almost 90% of the patients were taking vitamin D supplements.

In a small study published as a conference abstract, Ling et al. found reduced occurrence of OP in patients with PMR treated with GCs compared to non-users [19], but this study lacks appropriate adjustment for confounders. Since GC use was treated as a dichotomous variable (“yes”/”no”), these results are of doubtful significance [19].

Most other studies investigating bone density and GCs in vasculitis are older. Dolan et al. also compared patients with PMR taking GCs to non-users in 1997 [20], finding that users had more active disease and decreased bone density at the spine at 24 months. However, statistical comparisons were made without adjustments (e.g., for the observed higher disease activity), so these results are not very helpful in understanding the effect of GCs on bone density in PMR. In a study from 2000, Haugeberg et al. found no significant differences in bone density between current GC users and non-users [21].

A cross-sectional study of 99 patients with ANCA-associated vasculitis reported, in 2002, a negative correlation between cumulative GC doses and bone density (expressed as a Z-score), but did not assess the role of current GC intake [22]. The differences in these findings compared to our study might be related to insufficient adjustment for confounders in the former study. The detailed clinical data available in Rh-GIOP allow for comprehensive adjustment, including risk factors that might not even have been known two decades ago (e.g., PPI). Furthermore, the treatment options for ANCA-associated vasculitis were limited at the time (e.g., rituximab was FDA-approved for granulomatosis with polyangiitis in 2011). The few treatment options might have led to poorer disease control and the use of higher doses of GCs, which, in turn, might have worsened the bone-related AEs of GCs.

We hypothesized that attenuation of inflammation might alter the effect that GCs exert on bone density. A highly cited publication found that increased levels of CRP generally increase the risk of non-traumatic fractures [23]. However, we found no evidence in our analyses that CRP is associated with the bone density of patients with PMR, GCA or other vasculitides. Apparently, other confounders may play a more important role in this regard.

Across all analyses, lower BMI and PPI intake were consistently associated with lower bone density, and may have a larger effect on bone density than inflammation. While the influence of BMI on bone density is well known, the risk associated with PPI intake is often underestimated. We only found a single study investigating this association in vasculitis. This study of patients with ANCA-associated vasculitis found a higher risk of fractures in PPI users compared to histamine-2 receptor antagonist users, which is in line with our findings across all vasculitides [24]. Because patients receiving higher doses of GCs commonly receive PPIs to mitigate the risk of gastroduodenal ulceration, observational studies investigating GC effects on bone should always adjust for PPI intake.

Our study has several limitations. Since it is a cross-sectional analysis, association, but not causation, is demonstrated. Reproduction and confirmation of the results from the longitudinal analyses of our ongoing Rh-GIOP and other cohorts would strengthen our findings. While we conducted comprehensive adjustments for known and expected risk factors, even accounting for potential interactions, it is possible that unknown confounders might bias our study. Finally, we only measured bone density. It has been suggested that GCs might increase the risk of fracture—in part—independently of bone density [25]. The number of fractures in our cohort is currently too small to draw such conclusions. We decided against transforming T-scores into a binary variable (OP: “yes/no”), as information would be lost from a statistical point of view [10].

A strength of this study is the availability of data on multiple relevant patient and clinical variables that are essential to a well-conducted cohort design, which enables adjustments in analytic models. Only a minor proportion of data (5%) was missing. Another strength is the substantial number of patients enrolled, including those with PMR and rare diseases in the form of vasculitis. Bone density was consistently measured with the same equipment by the same operators, reducing inter-test variability. Finally, we modelled GC intake in several ways to account for possibly differential effects and non-linear relationships.

In summary, in patients with vasculitis or PMR treated at a tertiary care university hospital, OP seems to be GC-*associated,* rather than GC-*induced*. Low BMI and PPI intake as modifiable risk factors should be given more attention. Vitamin D supplementation, which was routinely used in our cohort, might mitigate damage to the bone. Further studies investigating GCs and bone density should adjust for concurrent PPI intake. We found no evidence that the net effect of GCs in bone density is mediated by suppression of inflammation. Confirmation of our findings is needed in longitudinal analyses of Rh-GIOP and other well-conducted cohort studies.

## Figures and Tables

**Table 1:** Description of main statistical models.

Model No.	Dependent Variable	Main Independent Variable
1	Minimum T-score	Current GC dose, continuous
2		Current GC dose, categorical
3		Cumulative GC dose, continuous
4		Cumulative GC dose, categorical

GC, glucocorticoid.

**Table 2:** Adjustment specifications for multivariable linear regression models.

**Potential Confounders Included in Multivariable Regression Models**
Age
Sex
Type of vasculitis
Smoking status (current, former, no smoking)
Body mass index
History of osteoporotic fractures (yes/no)
Family history of osteoporotic fractures (yes/no)
Alcohol consumption (none, irregular/infrequent, occasional, frequent)
Menopause (yes/no)
History of vertebral fractures (yes/no)
Health Assessment Questionnaire (HAQ)
Alkaline phosphatase
Gamma-glutamyltransferase
Proton pump inhibitor use (yes/no)
C-reactive protein
Disease duration
Bisphosphonate use (yes/no)
Denosumab use (yes/no)
Vitamin D deficiency (no deficiency/subclinical/clinically relevant)
Tocilizumab intake (yes/no)
Cumulative duration of GC use ^1^
**Interaction Terms Included in Multivariable Regression Models**
CRP: Tocilizumab intake
CRP: GC ^1^
GC: Type of vasculitis ^1^
GC: Disease duration ^1^
GC: Cumulative duration of GC use ^1^
GC: HAQ ^1^
Menopause: Sex

^1^ Glucocorticoids (GCs) modelled as either current or cumulative, and continuous or categorical, depending on the model.

**Table 3:** Demographics.

Overall	*n* = 198
Age, years *n (%)*	67.69 (11.4)
Sex (male) *n (%)*	66 (33.3)
Type of vasculitis *n (%)*	
Polymyalgia rheumatica	71 (35.9)
Giant cell arteritis	51 (25.8)
c-ANCA-associated vasculitis *	36 (18.2)
p-ANCA-associated vasculitis **	21 (10.6)
Takayasu’s arteritis	8 (4.9)
Undifferentiated vasculitis	6 (3.0)
Polyarteritis nodosa	4 (2.0)
Cogan’s syndrome	1 (0.5)
Disease duration, years *mean (SD)*	5.31 (6.33)
Glucocorticoid intake	
Current intake *n (%)*	173 (87.4)
Current dose, mg/d *mean (SD)*	30.79 (67.5)
0	25 (12.6)
0.01–4	27 (13.6)
4.01–7.5	49 (24.7)
7.51–30	53 (26.8)
>30	44 (22.2)
Cumulative dose, g *mean (SD)*	13.21 (22.15)
Cumulative duration of use, years *mean (SD)*	4.80 (6.61)
Disease-modifying anti-rheumatic drugs *n (%)*	118 (59.6)
Conventional synthetic	87 (43.9)
Biological	31 (15.7)
Minimum T-score *mean (SD)*	−1.74 (0.9)
Osteoporosis by DXA	39 (19.7)
Family history of osteoporosis *n (%)*	27 (19.1)
Family history of osteoporotic fracture	18 (12.8)
Prior osteoporotic fracture *n (%)*	65 (32.8)
Prior vertebral fracture *n (%)*	19 (9.6)
Anti-osteoporotic therapy *n (%)*	
Bisphosphonates	29 (14.6)
Denosumab	6 (3)
25-OH vitamin D3 deficiency *n (%)*	
Subclinical (25–50 nmol/L)	9 (4.5)
Clinically relevant (<25 nmol/L)	5 (2.5)
Vitamin D supplementation *n (%)*	176 (88.9)
Calcium supplementation *n (%)*	8 (4.0)
C-reactive protein, mg/L *mean (SD)*	13.15 (26.05)
Body mass index *mean (SD)*	26.44 (4.44)
Smoking status *n (%)*	
Never	99 (50.8)
Former smoker	78 (40.0)
Current smoker	18 (9.2)
Alcohol consumption *n (%)*	
None	83 (43.0)
Irregular/infrequent	81 (42.0)
Occasional	24 (12.4)
Frequent	5 (2.6)

* Includes granulomatosis with polyangiitis. ** Includes eosinophilic granulomatosis with polyangiitis and microscopic polyangiitis.

**Table 4:** Results of adjusted multiple linear regression analyses with minimum T-score as the dependent variable and current and cumulative glucocorticoid (GC) intake as continuous or categorical predictors.

	Slope *β*	97.5% CI	Adjusted R²	*p*
**Current GC dose (continuous)**	−0.01	−0.02 to 0.01	22.70%	0.49
**Current GC dose (categorical)**			20.00%	
0 mg/d (reference)	-	-		-
>0–4 mg/d	−0.03	−1.11 to 1.04		0.96 *
>4–7.5 mg/d	0.32	−0.69 to 1.32		0.53 *
>7.5–30 mg/d	−0.14	−1.09 to 0.81		0.77 *
>30 mg/d	0.21	−1.28 to 1.69		0.78 *
**Cumulative GC dose (continuous)**	0.01	−0.04 to 0.07	23.10%	0.59
**Cumulative GC dose (categorical)**			26.00%	
>1.5–5.7 g	−0.30	−1.05 to 0.44		0.42 *
>5.7–15.5 g	0.38	−0.43 to 1.20		0.35 *
>15.5 g	−0.00	−1.29 to 1.29		1.00 *

* Comparison with reference group.

**Table 5:** Sensitivity analyses.

	Polymyalgia Rheumatica Excluded	Lumbar Spine Ts as Dependent Variable	Right Femoral Neck Ts as Dependent Variable	Left Femoral Neck TS as Dependent Variable
*p*				
Current GC dose (continuous)	0.90	0.54	0.92	0.41
Current GC dose (categorical)	≥0.47	≥0.47	≥0.44	≥0.06
Cumulative GC dose (continuous)	0.31	0.71	0.36	0.30
Cumulative GC dose (categorical)	≥0.12	≥0.33	≥0.11	≥0.16

GC, glucocorticoid; Ts, T-score.

## Data Availability

Data are not publicly available.

## Supplementary Materials

The following supporting information can be downloaded at: https://www.mdpi.com/article/10.3390/cells11030536/s1; Table S1: Data collected at each patient visit (by questionnaire and measurements).
