# Supplementary material for: Glucocorticoids Are Not Associated with Bone Mineral Density in Patients with Polymyalgia Rheumatica, Giant Cell Arteritis and Other Vasculitides—Cross-Sectional Baseline Analysis of the Prospective Rh-GIOP Cohort
Source: Cells. 2022 Feb 4;11(3):536. doi: 10.3390/cells11030536 (PMC8834394; doi:10.3390/cells11030536)
Supplement: Supplementary file 1 [file cells-11-00536-s001.zip › cells-1556803-supplementary.pdf]

Supplementary Table S1. Data collected at each patient visit (by questionnaire and measurements) .

Demographics and general information	Age, sex, BMI, smoking status, alcohol consumption, type and frequency of physical exercise, exposure to direct sun light, daily calcium intake, use of care services, socio-economic status
Description of GC therapy	Current GC dose, mean daily GC dose, cumulative (lifetime) GC dose*, and duration of GC therapy
Description of underlying disease	Onset of disease, comorbidities, selected patient reported outcomes (Pain NAS, HAQ), past and current antirheumatic drugs
General bone-relevant parameters	Vitamin D and calcium supplementation, treatment with anti-osteoporotic drugs, treatment with drugs having a known or potential impact on bone (e.g., proton pump inhibitors)
Clinical bone-relevant parameters	Family history of osteoporosis/osteoporotic fractures, frailty assessment (timed-up and go-test, chair-rising-test, tandem stand), back pain, prior low-trauma vertebral and non-vertebral fractures**, date of fracture, management of fractures, fracture sequalae, weight loss, loss of height, past falls, risk assessment of falls, back pain, menarche/menopause/pregnancies/ lactation / past use of hormone-based contraceptives
Technical bone-relevant parameters	Routine laboratory parameters such as calcium, phosphate, vitamin D levels (1,25 and 25), iPTH, bone alkaline phosphatase, crosslinks and other, BMD / T-Score measured by DXA, TBS

BMI, body mass index; ESR erythrocyte sedimentation rate; CRP, c-reactive protein; BMD, bone mineral density; GC, glucocorticoid; iPTH, intact parathyroid hormone; DXA, Dual Energy X-ray Absorptiometry; TBS, Trabecular Bone Score. When patients were not able to provide full or detailed information, patient charts were used to complement the investigated parameters. *Cumulative Glucocorticoid-dose was meticulously calculated from patients' self-reported dose and duration of glucocorticoid therapy with the help of supplemental data retrieved from patient charts. **History of fractures was self-reported and verified from patient charts if available. Fractures were adjudicated under osteoporotic fractures when having occurred due to inadequate trauma or fall from standing height.
